# Plasticity Through Canalization: The Contrasting Effect of Temperature on Trait Size and Growth in *Drosophila*

**DOI:** 10.3389/fcell.2018.00156

**Published:** 2018-11-20

**Authors:** Jeanne M. C. McDonald, Shampa M. Ghosh, Samuel J. L. Gascoigne, Alexander W. Shingleton

**Affiliations:** ^1^Department of Biology, Lake Forest College, Lake Forest, IL, United States; ^2^Kalinga Institute of Industrial Technology (KIIT), Bhubaneswar, India; ^3^Department of Biological Sciences, University of Illinois at Chicago, Chicago, IL, United States

**Keywords:** Size control, thermal plasticity, temperature-size-rule, cell proliferation, imaginal disks, body proportion, morphology, canalization

## Abstract

In most ectotherms, a reduction in developmental temperature leads to an increase in body size, a phenomenon known as the temperature size rule (TSR). In *Drosophila melanogaster*, temperature affects body size primarily by affecting critical size, the point in development when larvae initiate the hormonal cascade that stops growth and starts metamorphosis. However, while the thermal plasticity of critical size can explain the effect of temperature on overall body size, it cannot entirely account for the effect of temperature on the size of individual traits, which vary in their thermal sensitivity. Specifically, the legs and male genitalia show reduced thermal plasticity for size, while the wings show elevated thermal plasticity, relative to overall body size. Here, we show that these differences in thermal plasticity among traits reflect, in part, differences in the effect of temperature on the rates of cell proliferation during trait growth. Counterintuitively, the elevated thermal plasticity of the wings is due to canalization in the rate of cell proliferation across temperatures. The opposite is true for the legs. These data reveal that environmental canalization at one level of organization may explain plasticity at another, and vice versa.

## Introduction

Temperature impacts multiple aspects of biology through its influence on the rates of almost all biological processes. Endotherms are able to mitigate these effects through their ability to generate their own heat and maintain a more-or-less constant core body temperature. Body temperature in ectotherms, in contrast, fluctuates with the thermal environment, which has a much more significant impact on their phenotype. While temperature has obvious effects on ectotherm physiology, particularly its effect on the rate of biochemical reactions and biological processes, it also impacts ectotherm morphology. Specifically, most ectotherms mature at a smaller size when reared at a higher temperature, a phenomenon so ubiquitous it is referred to as the temperature size rule (TSR) (Ray, 1960; Atkinson, 1994). While almost 83% of ectotherm species obey the TSR (Atkinson and Sibly, 1997), we have a remarkably poor understanding of its proximate (mechanistic) and ultimate (evolutionary) causes. Indeed, there is still debate as to whether the TSR is a consequence of selection for mechanisms that reduce body size at higher temperatures or due to the near-universal effects of temperature on the biophysical processes that regulate the rate of growth and development (Atkinson and Sibly, 1997; Angilletta and Dunham, 2003; Angilletta et al., 2004). That is, it is unclear whether or not the TSR is an adaptation.

Perhaps the most compelling evidence that the TSR is an adaptation comes from the observation that different traits within the same body obey the TSR to different degrees. In *Drosophila* *melanogaster*, for example, the wings are unusually thermally plastic, such that their size is much more sensitive to changes in developmental temperature than the body as a whole (Azevedo et al., 2002; Shingleton et al., 2009). A consequence of this is that flies reared at lower temperatures have proportionally larger wings. One compelling hypothesis for this phenomenon is that, because wing-stroke frequency is reduced at lower temperatures, flies require proportionally larger wings to fly, with a correspondingly reduced wing-loading. This hypothesis is supported by data showing that *Drosophila* reared at low temperatures have better cold-flight performance than *Drosophila* reared at high temperatures (Frazier et al., 2008). In contrast to the wings, other *Drosophila* traits, in particular the front legs and male genitalia, are thermally implastic (Azevedo et al., 2002; Shingleton et al., 2009). The reduced plasticity of the male genitalia is consistent with the observation that male genital size in *Drosophila*, and indeed most arthropods, shows very low variability within a species (Eberhard et al., 1998; Eberhard, 2009). In *Drosophila*, for example, the male genitalia also show reduced nutritional plasticity, and are less genetically variable than other traits (Shingleton et al., 2009; Dreyer and Shingleton, 2011). This low variability appears to be related to the use of the male genitalia in species recognition: females are thought to use genital traits to recognize conspecifics (Eberhard et al., 1998; Eberhard, 2009), and female *Drosophila* resist reproducing with males with inappropriately sized genitalia (Frazee and Masly, 2015). Thus, the thermal sensitivity of both the wings and the male genitals appears to be subject to selection for a change in their plasticity relative to that of the body as a whole.

If selection is able to modify the thermal plasticity of individual traits, then it must be doing so by impinging on the developmental mechanisms that regulate trait size in response to temperature, which are currently unknown (although see Li and Gong, 2015). Adult morphological traits, such as the wing, leg and genitalia, are generated from imaginal disks that grow internally during larval development. Like the body as a whole, adult trait size is regulated by the size of the disk at attainment of critical size, the duration of the disk’s terminal growth period (TGP) (the period between the attainment of critical size and the cessation of growth), and the rate of growth during the disk’s TGP (Shingleton et al., 2008; Nijhout et al., 2014). Thermal plasticity of trait size could be regulated by the effect of temperature on one or all of these factors. Since both attainment of critical size and duration of the TGP are systemically regulated by circulating hormones (Nijhout et al., 2014; Gokhale and Shingleton, 2015), thermal changes in critical size and the body’s TGP will also influence the size of the disks at critical size and the duration of their individual TGPs. These regulators of trait size are unlikely to account for *relative* differences in the thermal plasticities of individual traits because they are systemic in nature. Differences among traits in thermal sensitivity are therefore likely to be mediated by the differences in the effect of temperature on trait growth rate.

An added nuance, however, is that during metamorphosis of the pupa there is substantial change in cell size as the imaginal disks evert and differentiate into their final structures. Imaginal disk cells are very small: the average apical area of a wing disk cell is 4.92 μm^2^ at 25°C (Wartlick et al., 2011). In contrast, the average apical area of an adult wing cell is 140 μm^2^ at 25°C (data from Shingleton et al., 2009 and this study). Differences among traits in their thermal sensitivity could therefore reflect differences in their final cell size, and arise during metamorphosis rather than during larval development. If this were the case, disk growth rate prior to metamorphosis could be equally thermally sensitive across traits.

In this paper. we test the hypothesis that differences in the thermal sensitivities of different morphological traits in *Drosophila* arise through differences in the effect of temperature on the rate of growth – specifically cell proliferation – in different imaginal disks. Using clonal analysis, we measured the cell doubling time (CDT) in the wing and leg imaginal disk of third instar larvae reared at 17 and 25°C. We also measured imaginal disk cell number at critical size and at pupariation at the two temperatures. Combining these data with data from previous studies showing the effect of temperature on developmental time, we built a mathematical model of disk growth at different temperatures. Our model indicates that, counterintuitively, the elevated thermal plasticity of wing size relative to leg size arises through a reduction in the thermal plasticity of cell proliferation in the wing imaginal disk relative to the leg imaginal disk.

## Materials and Methods

### Fly Stocks

Trait plasticity and disk growth rate: *Samarkand* (*Sam*). Clonal analysis: *y[1] w[^∗^]; P{w[+mC] = AyGAL4}25 P{w[+mC] = UAS-GFP.S65T}Myo31DF[T2]* (BDSC: 4411), *P{ry[+t7.2] = hsFLP}12, y[1] w[^∗^]; sna[Sco]/CyO* (BDSC: 1929).

### Thermal Plasticity

**FIGURE 1:**
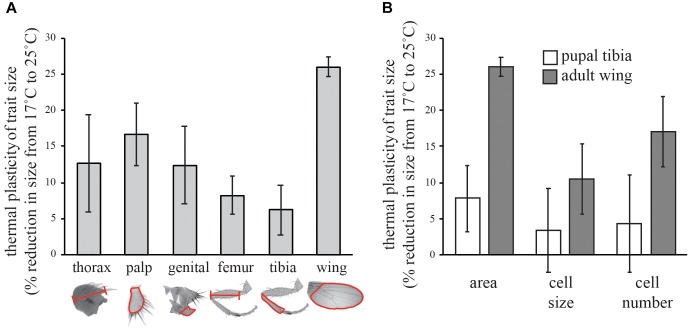
Effect of temperature on the trait size in *Drosophila.* **(A)** Percent reduction in the size of different morphological traits from 17 to 25°C. **(B)** Percent reduction in the cell size and cell number of the adult wings and pupal tibia (stage 9) from 17 to 25°C. All error bars are 95% confidence intervals of the mean.

To determine the relative thermal plasticity of different traits, *Samarkand* flies reared from egg to adult on standard cornmeal-molasses medium at 17 and 25°C. The medium comprised: 390 g molasses, 245 g yellow cornmeal, 50 g yeast, 27 g carrageenan, 12 g propionic acid, 2.5 g methyl paraben and 25 ml ethanol in 4.25 l water. The wing, first leg, maxillary palp, and genital arch from the right side of adult male flies, and wing cell size and cell number, were measured from 25 males at each temperature, using previously described methods (Shingleton et al., 2005, 2009). The measurements taken are shown outlined in red in Figure 1A. Wing cell size was estimated by counting the number of trichomes in a 100 × 100 μm square between veins IV and V of the dorsal wing blade and dividing the area of the square (10,000 μm^2^) by the trichome count. Wing cell number was estimated by dividing the wing area by cell size. Leg measurements were squared prior to analysis. Leg cell size and number was calculated from legs dissected at pupal stage 9 (Bainbridge and Bownes, 1981), when the leg cells are still visible through the cuticle (Azevedo et al., 2002). Pupal legs were dissected in methanol, washed in PBT (PBS + 0.2% Tween 20), mounted in Vectashield (Vector Laboratories, Burlingame, CA, United States) with DAPI, and imaged using standard epifluorescence microscopy. We collected 20 legs at 25°C and 18 legs at 17°C. Leg cell size was estimated by counting the number of DAPI-stained nuclei in five 25 × 25 μm squares randomly spaced across the surface of the tibia, and dividing the area of the square (625 μm^2^) by the average nuclei count. Leg cell number was estimated by dividing tibia area (outlined in Figure 1A) by average cell size. To test for a difference in thermal plasticity between the wing and leg size we tested whether there was an interaction between the effect of temperature and trait identity on size, using the linear model *S_ijk_* = *T_i_*+ *D_j_*+ *T_i_*^∗^*D_j_* + *e_ijk_* where *S* is log(trait size), *T* is temperature, *D* is trait identity *e* is error (subscripts are levels within factors). Percent reductions in trait size, cell size and cell number from 17 to 25°C were calculated on the untransformed data.

### Clonal Analysis

Clones were induced using the flp-out system and marked using GFP (Ito et al., 1997). Flies were of the genotype *hsflp; AyGAL4 UAS-GFP*. In these flies, heatshock-induced expression of flippase excises an FRT-bound cassette that separates an actin-promotor from GAL4. Excision of the cassette, which occurs randomly in dividing cells, allows the actin-promotor to drive constitutive expression of GFP in the cell and its daughters. A complete description of the methodology is provided by Ito et al. (1997). Larvae were reared at two temperatures: 17 and 25°C. Larvae were heat-shocked at 37°C for 1.5, 46 h after hatching for 25°C larvae and 100 h after hatching for 17°C larvae (just before ecdysis to the third larval instar at each temperature). Clones were left to develop for 48 and 96 h at 25 and 17°C, respectively, before seven larvae at each temperature were sacrificed and their wing and first leg imaginal disks were dissected and fixed. Between one and seven clones in each disk were imaged using standard methods. We calculated the rate of cell proliferation for each clone as log(N)/t where N is the number of cells in each clone and t is the age of the clone. The effect of temperature and disk type (fixed effect) on the rate of cell proliferation was tested using the linear mixed effect model *R_ijkl_* = *T_i_*+ *D_j_*+ *T_i_^∗^D_j_*+ *F_k_*+ *e_ijkl_*, where *R* is proliferation rate, *T* is temperature (fixed effect), *D* is disc type (fixed effect), *F* is fly ID (random effect) and *e* is error.

### Imaginal Disk Cell Number

Adult *Samarkand* females were allowed to oviposit on standard food plates for 4 h at 25°C. The food plates were then transferred to either 17 or 25°C and the larvae were allowed to develop to the third larval instar. The wing and first leg imaginal disks were dissected at attainment of critical size: 1.06 ± 0.05 mg at 17°C and 0.86 ± 0.05 mg at 25°C (Ghosh et al., 2013). Disks were dissected in PBS, fixed in 4% paraformaldehyde in PBS, washed in PBT, and mounted in Vectashield with DAPI. Each imaginal disks was imaged and its area was calculated as a measure of disk size. Cell size was estimated by counting the number of DAPI-stained nuclei in three 25 × 25 μm squares randomly spaced across the surface of the disk, and dividing the area of the square (625 μm^2^) by the average nuclei count. Cell number was calculated by dividing disk size by cell size, and multiplying by two (since the imaginal disks are bi-layered). We measured 10 and 8 leg disks at 17 and 25°C, and 13 and 5 wing disks at 17 and 25°C, respectively.

All statistical analyses were conducted in *R*, and the data are available for download on Dryad^1^.

## Results

### Wing Size Is Unusually Thermally Plastic

Consistent with published results (Azevedo et al., 2002; Frazier et al., 2008; Shingleton et al., 2009), we found that wing size was significantly more thermally plastic than leg size (as measured by the femur and tibia of the first leg) or the size of any other trait we measured (Figure 1A). For the adult wing, the change in size was due to a change in both cell size and cell number (Figure 1B). Cell size was reduced by 10% and cell number was reduced by 17% from 17 to 25°C, generating a 26% reduction in wing size. In contrast, in the stage 9 pupal tibia, neither cell size nor cell number was significantly affected by temperature (Figure 1B).

### The Rate of Cell Proliferation in the Wing Is Less Thermally Sensitive Than in the Leg

**FIGURE 2:**
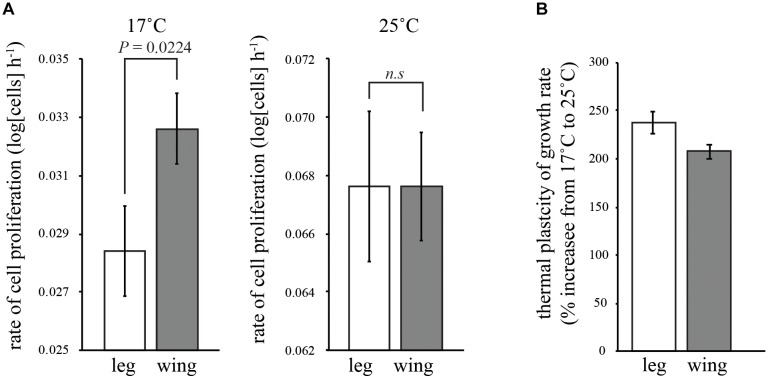
Effect of temperature on the rate of cell proliferation in the wing and first-leg imaginal disks. **(A)** Cell proliferation in the leg is significantly slower than the wing at 17°C but not significantly different from the wing at 25°C. **(B)** Consequently, temperature had more of an effect on the rate of cell proliferation in the leg than in the wing. Error bars are 95% confidence intervals.

In both the leg and the wing imaginal disks, the rate of cell proliferation decreased with temperature (Table 1 and Figure 2A). However, while the rate of proliferation was the same in both disks at 25°C (Tukey HSD, *P* > 0.05), proliferation was significantly slower in the leg than the wing at 17°C (Tukey HSD, *P* < 0.05) (Figure 2A). Correspondingly, the rate of cell proliferation was significantly more thermally plastic in the leg disk than in the wing disk (Table 1 and Figure 2B).

**Table 1:** Results of linear mixed-effect model for the effect of temperature and disk identity on the rate of cell proliferation.

Factor	*SS*	*MS*	NumDF^1^	DenDF^2^	*F*	*P*
Temp	0.03187	0.03187	1	96.101	1495.54	<0.0001^∗∗∗^
Disk	0.00013	0.00013	1	96.101	6.29	0.01382^∗^
Temp^∗^Disk	0.0001	0.0001	1	96.101	4.82	0.03047^∗^

*^*1*^Numerator degrees of freedom. ^*2*^Denominator degrees of freedom. ^∗∗∗^*P* < 0.0001 and ^∗^*P* < 0.05*.

### A Model of Thermal Plasticity of Wing and Leg Size in *Drosophila*

**FIGURE 3:**
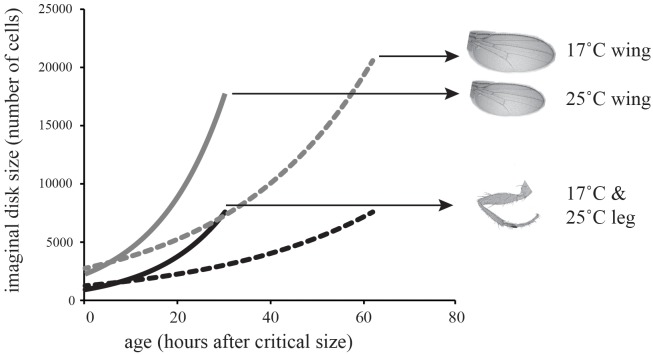
Model of the effect of temperature on imaginal disk growth through cell proliferation. The model predicts that wing cell number will be more plastic than leg cell number, matching the effect of temperature on cell number in the adult leg.

To determine whether differences in the thermal plasticity of cell proliferation in the wing relative to the leg could account for differences in their thermal plasticity of adult size, we modeled growth in the wing relative to the leg at 17 and 25°C (Figure 2B). In *Drosophila*, adult trait size is substantially controlled by the number of cells in the imaginal disks at the end of larval development and the beginning of metamorphosis. The decision to metamorphose is made early in the third larval instar when a larva reaches a critical size, attainment of which initiates the hormonal cascade that ends in pupation. Final trait size is therefore controlled by the size of the imaginal disks at the attainment of critical size, plus the amount of growth achieved between critical size and the cessation of growth, called the terminal growth period (TGP). This latter size increase is in turn controlled by the duration of the TGP plus the growth rate during the TGP. Imaginal disk growth is approximately exponential and so we can model final trait size as:

DF=DL·eR·T

where *D_F_* is final trait size, *D_L_* is trait size at critical size, *R* is the rate of growth, and *T* is the duration of the terminal growth period (Shingleton et al., 2008). To parameterize the model, we estimated the number of cells in the wing and first-leg imaginal disk at critical size at 17 and 25°C (*D_L_*); used our clonal analysis to estimate rate of cell proliferation in the two disks at both temperatures; and used published data on the duration of the TGP at both temperatures (Ghosh et al., 2013). For this latter parameter we used the time to pupariation from critical size, since cell proliferation in imaginal disks continues until pupariation (Simpson and Morata, 1981). All parameter values are shown in Table 2. Figure 3 shows the growth trajectory of the wing and leg imaginal disks at 17 and 25°C according to the model. The model predicts a 14% reduction in wing cell number from 17 to 25°C, while leg cell number is predicted to stay approximately the same. This fits well with the observed data (Figure 1).

**Table 2:** Parameters used to model imaginal disk growth at 17°C and 25°C (Figure 3).

Organ	Temperature	*D_L_*^1^	*R*^2^	*T*^3^	Final Cell Number	% Reduction
Wing	17°C	2717	0.0326	62	20531	13.7855746
	25°C	2326	0.0676	30	17701	
Leg	17°C	1287	0.0284	62	7487	-0.6815042
	25°C	991	0.0676	30	7538	

*^*1*^Cell number at critical size. ^*2*^Rate of cell proliferation [log(cell number) h^-1^]. ^*3*^Duration of the TGP (h) as reported in (Ghosh et al., 2013)*.

## Discussion

Our data reveal that temperature has more of an effect on wing size relative to the leg size in male *Drosophila*, and that this is, in part, due to differences in the thermosensitivity of cell proliferation in the growing wing relative to the growing leg. Specifically, temperature has less of an effect on the rate of cell proliferation in the growing wing relative to the leg; that is, cell proliferation is relatively thermally canalized (i.e., less plastic) in the wing compared to the leg. The apparently contradictory effects of temperature on adult trait size versus cell proliferation can be reconciled by also considering the effect of temperature on the duration of growth. At lower temperatures, the rate of cell proliferation declines, but the duration of growth increases. In the leg, the increase in growth duration from 25 to 17°C is offset by the decrease in growth rate, so that the net effect is only a small change in leg size with temperature. In contrast, because temperature has less of an effect on the rate of cell proliferation in the wing imaginal disk, the wing disks are able to better maintain their growth rate as temperature falls. Consequently, in the wing, the increase in growth duration from 25 to 17°C is *not* offset by the decrease in growth rate, so the wings end up growing larger at lower temperatures, increasing their thermal plasticity. The observation that temperature affects the rate of cell proliferation differently in different traits suggests that thermal plasticity is not simply due to general non-adaptive effects of temperature on the thermodynamics of growth and development, but that it is regulated through adaptive mechanisms.

Previous studies have also reported elevated thermal plasticity of the wing relative to other traits (Azevedo et al., 2002; Frazier et al., 2008; Shingleton et al., 2009). We found that the thermal plasticity of the wing was primarily due to changes in cell number, although there was a substantial effect of temperature on cell size. Results from previous studies suggest that the relative contribution of changes in cell size *vs.* cell number for thermal plasticity of wing size may vary across genotypes, and populations (Cavicchi et al., 1985; Partridge et al., 1994; Azevedo et al., 2002).

Further, there are also likely sex-specific differences in the relative contribution of these parameters to size plasticity: a similar reduction in wing cell number with an increase in temperature has been previously reported in male outbred flies, but not in females (Partridge et al., 1994). Our study was conducted only on males.

Our simple model of growth in wing and leg imaginal-disks demonstrates that thermal canalization of the rate of cell proliferation in the wing relative to the leg is sufficient to explain differences in the thermal plasticity of their final size. Nevertheless, the model is incomplete and only approximates published data on growth of the wing imaginal disk. For example, growth of the imaginal disks is not simply exponential but follows more of a Gompertz or logistic function, slowing at attainment of critical size and further slowing as the larva approaches pupariation (Bryant and Levinson, 1985; Nijhout and German, 2012). Further, our model predicts final wing cell number at 25°C to be 17,700, while previous studies have estimated wing cell number to be c.45,000–50,000 (Martin, 1982; Bryant and Levinson, 1985). There are a number of possible explanations for this. First, our measurement of the rate of cell proliferation in the wing disk at 25°C (0.068) is lower than previously reported [0.075–0.11 at 25°C (Martin, 1982; Bryant and Levinson, 1985)]. This low proliferation rate may be caused by a heat-shock induced delay in cell division (Pezzoli et al., 1986). Second, our estimate of wing cell number at critical size – 2326 cells at 25°C – is based on counting nuclei in mounted disks using a microscope. Previous studies, which counted dissociated cells, suggest that wing cell number is c. 2900–3700 at critical size (Martin, 1982; Bryant and Levinson, 1985). By adjusting the rate of cell proliferation at 25°C to 0.09, and the number of cells at critical size in the wing and leg disk to 3,300 (the average published values of these parameters), our model predicts final wing cell number to be 49,000. Further, by scaling the rate of cell proliferation at 17°C in the wing by 1.42, and the number of cells at critical size at 17°C by 1.33 (the factors by which the published estimates at 25°C differ from our measurements), the thermal plasticity of wing cell number remains 14% from 17 to 25°C, well within the 95% confidence interval of the observed effect of temperature on wing cell number (Figure 1B). Thus, while our model may not precisely predict the final number of cells in the wing, it does predict the thermal plasticity of wing size. Additional measurements of cell number and cell proliferation rates, ideally by counting dissociated cells, would help better parametrize the model.

Our data indicate that the difference in thermal plasticity among traits is regulated in part by the trait-specific effects of temperature on cell proliferation. We have, however, very little understanding of what signaling pathways regulate these differences. The underlying mechanisms are likely distinct from those that regulate nutritional plasticity, such as the IIS/TOR pathway, because the pattern of plasticity among traits in response to thermal variation is different from the pattern in response to nutritional variation (Shingleton et al., 2009). There are two views of the genes involved in regulating thermal plasticity (Scheiner, 1993; Via et al., 1995). The first view is that genes involved in trait morphogenesis are themselves sensitive to environmental variation, referred to as *allelic sensitivity*. The second is that there are genes that translate environmental variation into phenotypic variation, referred to as *genes for plasticity*. Several researchers have conducted screens for mutations that affects the thermal plasticity of *Drosophila* traits. Both (Carreira et al., 2013) and (Debat et al., 2009) looked at the effect of homozygous and heterozygous transposon insertions on the response of traits to temperature change. Most of the mutations affected thermal plasticity of trait size, wing size in the Debat et al. study, and wing, thorax, head and face size in the Carreira et al. study. Debat et al. targeted genes that are known to be involved in formation of the wing, and their data are consistent with the hypothesis that it is the allelic sensitivity of these genes that is responsible for thermal plasticity. Similarly, Cerreira et al. found very few genes that affected the plasticity of all traits, which also suggests that thermal plasticity is regulated by multiple genes involved in generating trait-specific morphology. Nevertheless, the limited scope of their screen [42 and 16 genes screened in Carreira et al. (2013) and Debat et al. (2009), respectively] makes it highly unlikely that they would have uncovered “genes for plasticity” that regulate thermal plasticity in general. More detailed comparisons of how the wing and the leg differentially respond to temperature should help uncover these general mechanisms, if they exist.

While our understanding of the cellular mechanisms of thermal plasticity is limited, the evolutionary mechanism to explain the elevated thermal plasticity of the wing has been better elucidated. At colder temperatures, *Drosophila* muscle contraction is impaired and wingbeat frequency is reduced. By increasing the wing:body size ratio, this reduction in wing-beat frequency can be compensated for by reducing wing loading. Consequently, flies that are able to proportionally increase wing size at lower temperatures relative to other traits have a selective advantage (Frazier et al., 2008). Globally, it has been well-documented that *Drosophila* populations at colder temperatures also possess larger wings relative to body size than those at warmer temperatures, even when reared at the same temperature (Azevedo et al., 2002; Klepsatel et al., 2014). This geographic difference in wing size is also likely due to the selective advantage of improved flight performance that larger wings confer at colder temperatures (Azevedo et al., 2002). However, whether the geographic variation in relative wing size is generated through the same developmental mechanisms as the plastic response to temperature is an open question.

Broadly, our study is consistent with the hypothesis that thermal plasticity is adaptive. If thermal plasticity were solely determined by biophysical constraints, we would not expect to see differences in the sensitivities of trait size to developmental temperature, particularly differences that can potentially contribute to fitness, as in the case of wings. Further, we have shown that elevated plasticity of a trait appears to be governed by canalization of an underlying cellular process, thus demonstrating that plasticity at one level of organization may be caused by canalization at another, and vice versa. More generally, further studies into the developmental mechanisms that underlie the elevated thermal plasticity of the wing will not only help explain how individual traits can modify their thermal plasticity, but will help reveal the developmental mechanisms that generate variation in body and trait size with temperature in general.

## Data Availability Statement

The datasets analyzed for this study can be found on Dryad.

## Author Contributions

AS and SMG devised the study and designed the experiments. JM, SMG, and AS analyzed the data. All the authors were involved in conducting the experiments and writing the manuscript.

## Conflict of Interest Statement

The authors declare that the research was conducted in the absence of any commercial or financial relationships that could be construed as a potential conflict of interest.

## Footnotes

1 https://datadryad.org/
